# Cancer-associated fibroblasts and its derived exosomes: a new perspective for reshaping the tumor microenvironment

**DOI:** 10.1186/s10020-023-00665-y

**Published:** 2023-05-22

**Authors:** Zhiwei Peng, Zhiwei Tong, Zihao Ren, Manping Ye, Kongwang Hu

**Affiliations:** 1 Department of General Surgery, First Affiliated Hospital of Anhui Medical University, Anhui Hefei, 230022 China; 2 Anhui Province Key Laboratory of Major Autoimmune Diseases, Anhui Institute of Innovative Drugs, School of Pharmacy, Anhui Medical University, Anhui Hefei, 230032 China; 3 Department of General Surgery, Fuyang Affiliated Hospital of Anhui Medical University, Anhui Fuyang, 236000 China

**Keywords:** Cancer-associated fibroblasts, Exosomes, Tumor microenvironment, Diagnosis, Therapy

## Abstract

Cancer-associated fibroblasts (CAFs) are the most abundant stromal cells within the tumor microenvironment (TME). They extensively communicate with the other cells. Exosome-packed bioactive molecules derived from CAFs can reshape the TME by interacting with other cells and the extracellular matrix, which adds a new perspective for their clinical application in tumor targeted therapy. An in-depth understanding of the biological characteristics of CAF-derived exosomes (CDEs) is critical for depicting the detailed landscape of the TME and developing tailored therapeutic strategies for cancer treatment. In this review, we have summarized the functional roles of CAFs in the TME, particularly focusing on the extensive communication mediated by CDEs that contain biological molecules such as miRNAs, proteins, metabolites, and other components. In addition, we have also highlighted the prospects for diagnostic and therapeutic applications based on CDEs, which could guide the future development of exosome-targeted anti-tumor drugs.

## Background

Cancer is a genetic disease characterized by an uncontrolled proliferation of malignant cells. According to an estimate by the International Agency for Research on Cancer, there were 19.3 million new cancer cases and approximately 10 million cancer deaths globally in 2020. The global cancer burden is also expected to increase by 47% in 2040 as compared with that in 2020 (Sung et al. 2021). In the past decades, the study of cancer pathogenesis, diagnosis, treatment, and prognosis has progressed rapidly. However, the concomitant problems of tumor metastasis, immunosuppression, and drug resistance in cancer treatment are still not very well understood. The transition from tumor cell-centered tumorigenesis to tumor microenvironment (TME)-based tumor progression models has led to an increased appreciation of the critical role of the TME in neoplasm growth. The TME is a complex interstitial system with several cellular and acellular components, including the extracellular matrix (ECM) and various cells, such as immune cells, cancer-associated fibroblasts (CAFs), and endothelial cells (Bejarano et al. 2021; Corn et al. 2020).

CAFs are a common cell type within the TME. They extensively communicate with other cells in the TME and produce several markers associated with clinical prognosis (Herrera et al. 2021; Song et al. 2021). They promote tumor progression and distant metastasis and inhibit immune infiltration by secreting various soluble factors (Arina et al. 2016). In addition, CAFs can reshape the ECM to form a physical barrier that hinders drug and immune cell infiltration into tumor tissues, thereby reducing the effects of anti-tumor therapies (Arandkar et al. 2018). Crosstalk between CAFs, tumor cells, and other stromal cells is partially accomplished by exosomes. Exosomes are endosome-originated extracellular vesicles (EVs) with a physical diameter of 40–160 nm (Kalluri and LeBleu 2020). Based on their diverse cell precursors, exosomes can be involved in various physiological or pathological processes. These include immune responses, cellular development, metabolic disease pathways, cardiovascular fitness changes, and processes associated with tumor progression (Kalluri and LeBleu 2020). In recent years, several studies have elucidated the underlying mechanisms whereby CAF-derived exosomes (CDEs) contribute to the maintenance of malignant tumor phenotypes. Therefore, a thorough understanding of the biological processes mediated by CDEs in the TME could provide insights for the future discovery of anti-tumor agents and the development of targeted cancer therapies. In this review, we have discussed the origin, definition, and tumor-promoting or -inhibitory functions of CAFs. Furthermore, we have discussed the functional role of CDEs in the TME and the mechanisms of interaction between CDEs and the other cells. Moreover, we have summarized the existing applications of CDEs in clinical diagnosis, targeted therapies, and prognosis evaluation. This could lead to the development of new ideas for further exosome-based clinical application in the field of oncology.

## Heterogeneous biological hallmarks of CAFs

Fibroblasts are the main cellular component of loose connective tissues. They are of mesenchymal origin and maintain tissue homeostasis by secreting collagen and various soluble cytokines (Li et al. 2021). In healthy tissues, fibroblast proliferation and metabolism are maintained at low levels; however, they can be activated and increased during tissue injury or other pathological processes. Fibroblasts repair tissues through wound healing and fibrosis. This helps sustain a self-healing process in the body (Sahai et al. 2020). However, these activated fibroblasts also participate in tumor progression in addition to performing physiological tissue repair.

**Fig. 1:**
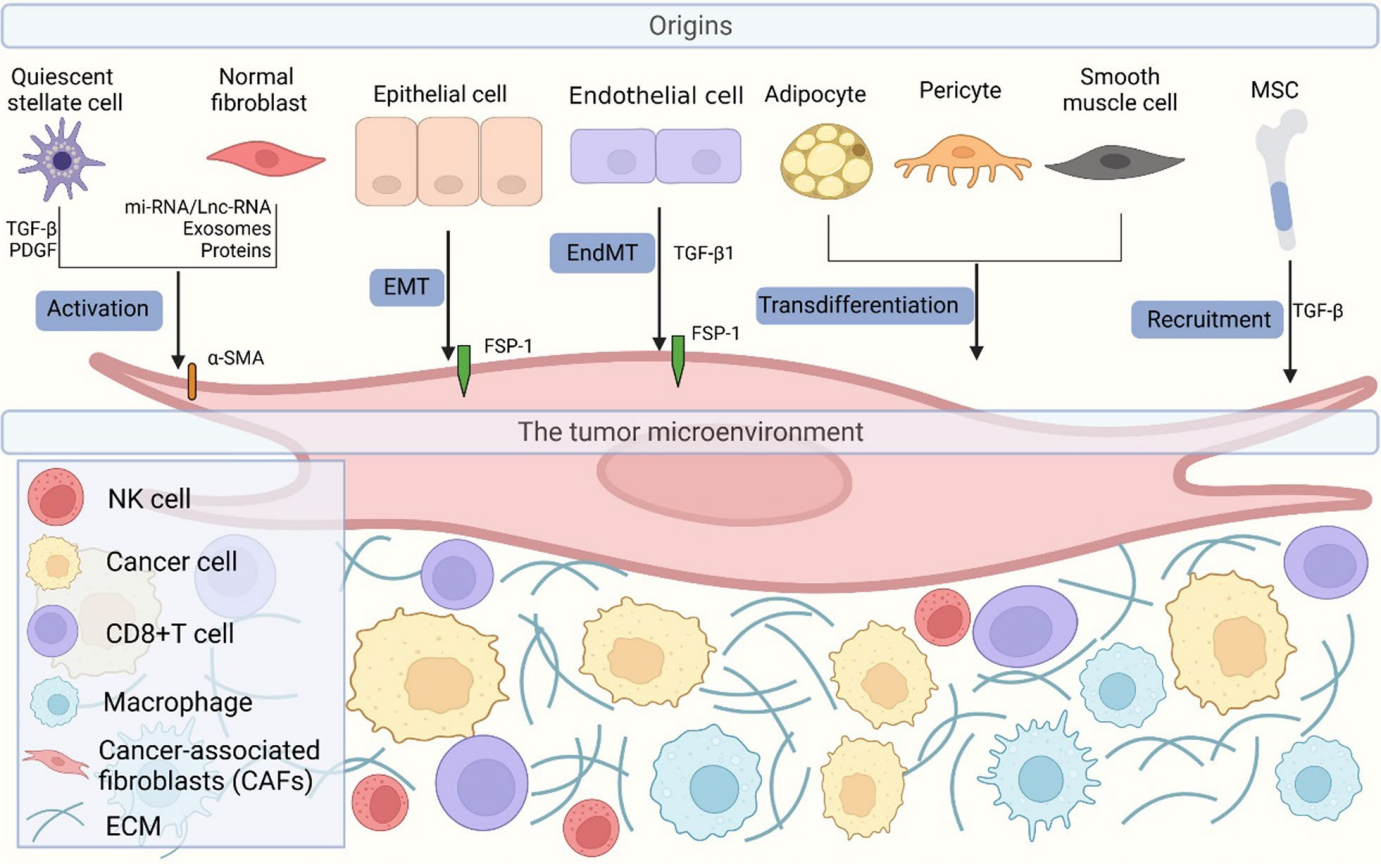
The origins of CAFs and its surrounding microenvironment components in tumor. CAFs are heterogeneous stromal cells abundantly existed in the TME, which have different cell sources activated by extensive TME-derived stimulants such as TGF-β, PDGF and other biological effect factors. Of which quiescent stellate cells and local resident normal fibroblasts, are the primary origin of CAFs; mesenchymal stem cells (MSCs), epithelial cells, adipocytes, pericytes, endothelial cells, and smooth muscle cells are also sources of CAFs within limited conditions. (The upper half of the figure portray the underlying origins of CAFs, while the lower half of the figure depict the tumor microenvironment components including various stromal cells and ECM around the CAFs)

With the rapid progression of tumor biological research, numerous studies have demonstrated that tumor occurrence and progression are also closely associated with the interactions between cancer cells and other cellular or non-cellular matrix components in the TME in addition to being related to the biological characteristics of the tumor itself. CAFs are the main stromal cells that are abundantly present in the TME. Numerous basic studies have reported that CAFs promote tumor growth, angiogenesis, tumor invasion, and metastasis. They also reshape the ECM, regulate tumor adhesion ability, and induce the generation of an immunosuppressive microenvironment (Sahai et al. 2020; Chen and Song 2019). However, some studies have also suggested that CAFs are composed of heterogeneous subsets, that have opposing functions in the TME. Alpha smooth muscle actin (α-SMA) depletion in a pancreatic cancer mouse model leads to the development of more aggressive tumors and shorter overall survival. This indicates that the α-SMA + fibroblast population consists of tumor suppressive fibroblasts (McAndrews et al. 2022). In addition, some studies have demonstrated that CD146- and versican (VCAN)-positive CAFs play an important role in tumor growth inhibition (Brechbuhl et al. 2017; Fanhchaksai et al. 2016). CAFs can be divided into distinct functional subgroups because of their multiple cell sources. CAFs mainly originate from in situ quiescent fibroblasts, such as pancreatic or hepatic stellate cells. However, CAFs can also originate from mesenchymal stem cells (MSCs), epithelial cells, pericytes, adipocytes, endothelial cells, and smooth muscle cells under limited conditions (Chen and Song 2019; Park et al. 2020) (Fig. 1).

Mechanistic studies have reported that tumor-derived stimulants induce the differentiation of normal fibroblasts or other cells into heterogeneous CAF subsets that are subsequently released by the tumors and their microenvironment. These stimulants include transforming growth factor-β (TGF-β) family ligands, lysophosphatidic acid, fibroblast growth factor (FGF), platelet-derived growth factor (PDGF), interleukin-1 (IL-1), interleukin-6 (IL-6), and granulin (Park et al. 2020). However, the specific origins and mechanisms of action of CAFs have not yet been clarified because of the complexity of the TME, However, tissue-specific origins and mechanisms of action of CAFs in some tumor types have been confirmed. Quiescent pancreatic stellate cells and hepatic stellate cells are the primary sources of CAFs, which can be transformed into α-SMA + CAFs via the activation of TGF-β, PDGF, and other tumor-derived stimuli. Therefore, these cells are regarded as precursors of pancreatic and liver cancer-derived CAFs (Omary et al. 2007; Yin et al. 2013). In addition to the activation of local resident fibroblasts, MSCs (another group of CAF precursor cells) are also recruited into tumor regions. These cells transdifferentiate into CAFs with tumor-promoting functions under the influence of tumor-derived cytokine and chemokine stimulations (Jung et al. 2013; Weber et al. 2015; Zhu et al. 2014). Additionally, CAFs can be produced by the conversion of other cell sources. During the process of fibrous tissue repair after injury, epithelial cells express fibroblast-specific protein-1 (FSP-1) after the epithelial-mesenchymal transition (EMT) and develop the phenotypic characteristics of fibroblasts. Similarly, endothelial cells stimulated by TGF-β1 undergo endothelial-to-mesenchymal transition and express the mesenchymal cell marker FSP-1 (Iwano et al. 2002; Zeisberg et al. 2007). Some recent studies have reported that adipocytes and smooth muscle cells can also be CAF sources. However, the specific mechanisms behind this need to be further explored (Okumura et al. 2019; Wikstrom et al. 2009).

While substantial progress has been made in the study of the origins of different subpopulations of CAFs and their related mechanisms, considering the functional and structural complexity of different TMEs, CAF origins have not been hitherto accurately elucidated. Notably, with the widespread application of single-cell sequencing technology, fibroblast subsets can be easily distinguished at the single-cell resolution level. Some studies have successfully identified CAFs with distinct functional phenotypes and determined the traces of their cell lineage (McAndrews et al. 2022). Hence, the development of treatment strategies targeting specific CAF subsets will further improve the efficacy of targeted treatments. It will also enable us to accurately predict their antitumor treatment effect and prognosis.

## Functional features of CAFs in tumor biology

### Tumorigenesis

CAFs interact with tumor cells or other stromal cells by secreting various cytokines, chemokines, metabolites, and exosomes that influence the indicators of tumor malignancy, such as cancer growth and invasion. (Li et al. 2021; Fiori et al. 2019).

Various CAF-secreted solute factors, such as IL-6, IL-33, TGF-β, stromal cell derived factor-1 (SDF-1), and CAF-derived cardiotrophin-like cytokine factor 1 (CLCF1) produce tumor-promoting signals that mediate the signal transduction of tumor cells or other cells in the TME (Song et al. 2021; Landskron et al. 2019; Qin et al. 2018; Tan et al. 2020; Wei et al. 2018). SDF-1 secreted by CAFs reportedly promotes the development of pancreatic cancer and gemcitabine resistance via SATB-1 expression upregulation in tumor cells (Wei et al. 2018). SATB-1 overexpression in pancreatic cancer cells facilitates a positive feedback loop that maintains CAF pro-cancer phenotypes. Similarly, TGF-β secreted by CAFs produces and maintains a similar pro-tumor positive feedback loop between the crosstalk of CAFs and cancer cells (Tan et al. 2020; Wu et al. 2022). Song et al. recently reported that the CAF-derived cytokine CLCF1 can enhance the secretions of CXCL6 and TGF-β in hepatocellular carcinoma (HCC) cells. This promotes tumor stemness and the N2 polarization process of tumor-associated neutrophils in an autocrine or paracrine manner (Song et al. 2021). They also reported that CXCL6 and TGF-β overexpression in cancer cells could activate the ERK1/2 signaling pathway in CAFs. This increases the secretion of CLCF1, thereby promoting HCC progression in a positive feedback loop-mediated pattern. This abovementioned research confirmed that CAFs have an extensive interaction with TME components and they synergistically promote tumor growth. Notably, the interaction is always reciprocal rather than unidirectional. Therefore, future therapies targeting CAFs should also identify tumor-derived specific soluble factors involved in CAF phenotype regulation in addition to considering the tumor-promoting signaling molecules of CAFs. Combination strategies that simultaneously target the reciprocal interactions between CAFs and tumor cells should be developed.

### Tumor metabolic reprogramming

Tumor metabolic reprogramming is a major hallmark of cancer. It plays an important role in tumor occurrence and development (Hanahan 2022). Previous studies have provided mainstream views of the central position of tumor cells in tumor metabolic patterns that lead to tumor progression and metastasis. However, the concept of TME has gained popularity. It has gradually demonstrated that various cellular types and acellular components contribute to tumor metabolic crosstalk in the TME, which could also result in tumor metabolic reprogramming (Martinez-Reyes and Chandel 2021; Wu et al. 2017). The nutrients in the TME are limited. Hence, the existence of various cell types, including cancer or stromal cells, could trigger a change in cellular metabolic patterns through several mechanisms that cells use to adapt to unfavorable conditions (Bian et al. 2020; Dey et al. 2021). Emerging evidence regarding the extensive metabolic crosstalk between CAFs and tumor cells has been uncovered. This allows researchers to determine CAF biology from a new perspective. Multiple studies have reported mutual metabolic communication between CAFs and tumor cells. CAFs change their metabolic patterns and produce corresponding soluble factors and metabolites that are taken up by the tumor cells. This promotes tumor cell growth and invasion. In addition, tumor cells may enhance metabolism-related gene expression, which could subsequently regulate the metabolic adaptation of CAFs, exacerbating tumor invasion (Martinez-Reyes and Chandel 2021; Zhu et al. 2020a, b).

According to recent studies, CAFs in the TME undergo various metabolic reprogramming processes, which affect tumor cell malignancy. These processes include glycolysis, lipid and amino acid metabolism, and oxidative phosphorylation (Zhu et al. 2020a, b; Demircioglu et al. 2020; Gong et al. 2020; Jin et al. 2022; Peng et al. 2021a, b; Zhang et al. 2020a, b, c). Zhu et al. demonstrated that tumor cells may target BCAT1 in CAFs through the TGF-β/SMAD5 pathway. They may also dictate the supply of various nutrients (such as alanine and glutamine) by CAFs to tumor cells under nutrient-deficient conditions. This is done via the internalization of ECM components and secretion of branched-chain amino acid components (Zhu et al. 2020a, b). CAFs also undergo lipid reprogramming in colorectal cancer (CRC). The intake of CAF-secreted small molecular metabolites by tumor cells potentiates the migration of tumor phenotype. Blocking this process reduces CAF-induced CRC cell migration. Hence, this has implications for the development of an optimal anti-tumor treatment strategy (Gong et al. 2020). CAF-derived soluble factors help maintain tumor cell nutrient intake via various metabolite secretions. They also affect tumor cell phenotypes through metabolic crosstalk. Focal adhesion kinase (FAK) depletion in CAFs triggers the release of the chemokine CCR1/CCR2 and enhances glycolysis in malignant cells (Demircioglu et al. 2020). Jin et al. reported that CAF-secreted CRMP2 facilitates ovarian cancer progression via the hypoxia-inducible factor (HIF)-1α-mediated glycolysis pathway (Jin et al. 2022). Studies have also demonstrated complex metabolic interactions between CAFs and other tumor cells. The recognition of extensive metabolic crosstalk in the TME has revealed the dilemma of whether a therapeutic strategy should target a particular metabolic pathway in CAFs or the cancer cells (Martinez-Reyes and Chandel 2021; Reina-Campos et al. 2017). Therefore, future studies should investigate treatment strategies targeting tumor–stroma-based metabolic interactions.

### Immunosuppressive microenvironment formation

Tumor immune escape is an important mechanism of tumor development and survival. It is also a major challenge in tumor immunotherapy (Hegde and Chen 2020). Various mechanisms of immune evasion have been reported in literature. Among these, immunosuppression has been recently highlighted. The specific mechanism behind this involves the down-regulation of tumor cell antigen presentation, increased surface inhibitory molecule expression, and immunosuppressive factor secretion (Bates et al. 2018). Clinical studies have revealed that tumor-killing immune cell infiltration in the TME is a key factor affecting tumor immunotherapy. Lower levels of immune cell infiltration are associated with a worse prognosis of patients with cancer (Ren et al. 2021). As a major TME component, CAFs can directly promote immunosuppression by increasing suppressive T lymphocyte infiltration and inhibiting effector T cell function (Costa et al. 2018; Feig et al. 2013; Kieffer et al. 2020; Wu et al. 2020). They can also reshape ECM components to form a physical barrier that directly inhibits anti-tumor immune cell recruitment (Glentis et al. 2017; Mao et al. 2021; Serrels et al. 2015).

Costa et al. observed that CAF-S1, a myofibroblast subset, attracts and retains CD4^+^CD25^+^ T lymphocytes by secreting CXCL12, OX40L, PD-L2, and JAM2 in breast cancer. CAF-S1 also promotes T lymphocyte proliferation and differentiation into CD25^High^FOXP3^High^ T cells through the secretion of B7H3, CD73, and DPP4. This enhances the regulatory T cell (Treg) immunosuppressive capacity (Costa et al. 2018). Studies using single-cell sequencing technology to further investigate CAF-S1 cell immunosuppressive function have reported that two CAF-S1 cell subsets (clusters 0 and 3 cells) are the main immunosuppressive stromal cells. Cluster 0 cells resist immune checkpoint blockade (ICB) treatment by upregulating PD-1 and CTLA4 levels in Tregs. This subsequently increases the number of CAF-S1 cluster 3 cells, thereby triggering immunosuppression via a positive feedback mechanism (Kieffer et al. 2020). Wu et al. observed that a subset of CAFs with immunomodulatory features can drive the dysfunction of cytotoxic T cells in triple-negative breast cancer (TNBC) (Wu et al. 2020). In a pancreatic ductal adenocarcinoma (PDAC) study, despite the finding that tumor-specific CD8^+^ T cells were observed in the tumor regions of PDAC mouse models, the mice did not respond to two immune checkpoint blockers (A-CTLA-4 and A-PD-L1) that promote the activity of T cells with tumor-inhibiting potential. This is because tumor regions are enriched with CAFs expressing fibroblast activating protein (FAP), which inhibits T lymphocyte aggregation by producing CXCL12 (Feig et al. 2013). CAFs also act on natural killer (NK) cells to promote their immunosuppressive functions. Francescone et al. demonstrated that PDAC progression is associated with NetG1^+^ CAF-driven immunosuppressive function, which can impede NK cell-mediated cancer cell depletion (Francescone et al. 2021). Several studies have reported that ECM remodeling is significantly correlated with tumor progression, metastasis, and immunosuppression (Mao et al. 2021; Acerbi et al. 2015). CAFs increase ECM stiffness by degrading the ECM structure, impeding the immune cells from entering the tumor areas, and reducing immune response generation in the TME (Glentis et al. 2017). Furthermore, CAFs form a chronic hypoxic microenvironment in the tumor area by accumulating matrix proteins (Gilkes et al. 2014). This subsequently promotes ECM stiffness via the HIF-1 pathway (Najafi et al. 2019). Moreover, hypoxia-induced vascular endothelial growth factor (VEGF) reduces the immune infiltration of T lymphocytes (Mao et al. 2021; Francesco et al. 2013), thereby maintaining the immunosuppressive microenvironment. CAFs can also trigger intracellular FAK activation by increasing ECM collagen density. This induces CD8^+^ T cell depletion and the recruitment of Tregs, myeloid-derived suppressor cells (MDSCs), and tumor-associated macrophages (TAMs). This recruitment contributes to immune response inhibition (Serrels et al. 2015; Bae et al. 2014). Hence, CDEs can directly or indirectly protect tumors by decreasing immune infiltration.

### Therapy resistance

Considering TME component biology and the extensive interactions among various stromal cells, tumor therapy resistance is a primary obstacle impeding the efficacy of anti-tumor therapy. The previous viewpoint was that tumor cells caused drug resistance. This has been gradually replaced by the detailed understanding of the mechanisms involving crosstalk between multiple TME components (Correia and Bissell 2012; Saw et al. 2022). CAFs trigger drug resistance through several mechanisms, and the relevant signaling pathways have been investigated in multiple studies.

Metabolic crosstalk between CAFs, tumor cells, and other stromal cells in the TME is an important factor in impeding drug sensitivity (Saw et al. 2022; Peng et al. 2022; Shi et al. 2022a, b; Zhang et al. 2020a, b, c). A recent study demonstrated that miR-20a was up-regulated in CAF-secreted exosomes. This promotes the tumorigenesis of non-small cell lung cancer (NSCLC) and explains cisplatin resistance. Further mechanistic studies have suggested that miR-20a could be delivered to tumor cells to inhibit the PTEN/AKT signaling pathway and suppress tumor growth. In contrast, targeting miR-20a could help restore the sensitivity of NSCLC to cisplatin (Shi et al. 2022a, b). In our previously published study, we identified two CAF subsets with different functional phenotypes in CRC. We also observed significantly increased numbers of tumor-promoting CAFs (named iCAFs) and decreased cellular metabolism in patients with CRC who underwent neoadjuvant chemotherapy. Notably, fatty acid metabolism did not decrease in the tumor region enriched with iCAFs. This suggests that the increased lipid metabolic activity caused by iCAFs in these patients may be a potential mechanism leading to chemotherapy resistance (Peng et al. 2022). A recent study has identified a new CAF subtype with highly glycolysis levels (named meCAFs) in pancreatic cancer. This subtype was related to immunotherapy response acquisition (Wang et al. 2021). Therefore, the mechanism by which different CAF subsets induce tumor drug resistance requires further exploration. In addition, CAFs can accelerate drug resistance by inhibiting the transport of the chemotherapeutic agents to tumor regions. This is because of ECM protein-formed physical barriers in the TME (Galindo-Pumarino et al. 2022; Kesh et al. 2020).

Extensive interactions between CAFs and immune cells are another possible mechanism for leading to drug resistance (Mao et al. 2021; Saw et al. 2022; Jenkins et al. 2022; Nicolas et al. 2022). In tumor immunotherapy, targeted drugs can reverse immunosuppressive effects by targeting immune checkpoints. Through complicated cell-cell communications, CAFs deplete the number of anti-tumor immune cells and impair their tumor-killing capacities. They also enrich the tumor immunosuppressive cells in tumor regions, resulting in ICB resistance in most tumor tissues. TAMs, the main immune cell components of tumor tissues, can be polarized into two different cell states: M1 polarization enhances macrophage anti-tumor effects and promotes tumor suppression. In contrast, M2 polarization promotes tumor invasion, metastasis, and immunosuppressive microenvironment formation (Mantovani et al. 2004). CAFs exert their immunosuppressive functions through different signaling pathways. They enrich monocytes and promote their reprogramming into M2-polarized TAMs (Chen et al. 2020; Cohen et al. 2017; Tan et al. 2018). In addition, CAFs activate neutrophils and induce tumor immunosuppression by releasing various cytokines such as CD66b, PDL1, IL8, TNFα, and CCL2. This is done via the IL6-signal transducer and activator of transcription 3 (STAT3)-PDL1 signaling cascades (Cheng et al. 2018).

### Tumor metastasis

Tumor metastasis is the main cause of death in patients with cancer. Tumor metastasis is caused by both tumor cells and their interaction with TME components (Kalluri 2016). Recently, CAFs have been observed to play an essential role in tumor metastasis (Eble and Niland 2019; Paauwe et al. 2018; Qiu et al. 2022); Ren et al. 2018a, b; Jungwirth et al. 2021). Studies have also demonstrated that interactions between CAFs and breast cancer cells, which are mediated by IL-32 and integrin β3 (ITGB3), play a crucial role in CAF-induced cancer metastasis. IL-32 is mainly derived from CAFs, whereas ITGB3 is upregulated during EMT in breast cancer cells. The specific binding of CAF-derived IL-32 to ITGB3 activates the p38 MAPK cascade signaling pathway in breast cancer cells. This upregulates EMT marker expression and promotes tumor metastasis. This binding significantly inhibits breast cancer metastasis and the EMT (Wen et al. 2019). In head and neck cancer (HNC), CAF-secreted IL-6 induces the expression of osteopontin and promotes tumor invasion and metastasis through the integrin αvβ3-NF-kappa B signaling pathway. The combination of osteopontin and IL-6 can be used as an improved diagnostic indicator and potential therapeutic target (Qin et al. 2018). Although CAFs promote tumor metastasis, metastatic microenvironments can also regulate CAF phenotypes by inducing CAF functional heterogeneity (Fang et al. 2018; Pan et al. 2021). There is a significant difference in prognosis between patients with PDAC who have concurrent liver and lung metastases and those who do not. Pan et al. (2021).

The CAF-promoting effect on tumor metastasis has been confirmed for different tumor types. However, the interaction between CAFs and other cellular components in the TME leading to metastasis remains unclear as yet. The mechanism by which different CAF subsets promote tumor metastasis also warrants further investigation.

### Tumor angiogenesis

Tumor angiogenesis is necessary for continuous tumor cell survival and progression in nutrient-limited microenvironments. Different stromal cells or non-cellular components, including the ECM, cytokines, and chemokines, have a significant impact on tumor angiogenesis (Palma et al. 2017; Jiang et al. 2020; Oshi et al. 2021). Previous studies have also suggested that CAFs secrete various pro-angiogenic cytokines such as VEGFa, FGF2, PDGFC, WNTs, and matrix metalloproteinases (Palma et al. 2017; Guo et al. 2021; Hou et al. 2010; Unterleuthner et al. 2020; Wan et al. 2021; Zhou et al. 2018). A unique hypoxic microenvironment combined with existing signaling molecules activates CAF-induced pro-angiogenesis function, mainly in a VEGF-dependent manner (Francesco et al. 2013; Zhou et al. 2018). Exosomal miR-155 secreted by melanoma cells promotes the expression of pro-angiogenic factors (VEGFa, FGF2, and matrix metalloproteinase) in CAFs by targeting the suppressor of cytokine signaling 1. This activates the Janus kinase 2/STAT3 signal transduction pathway (Zhou et al. 2018). In addition, Francesco et al. (2013).

CAFs can also accelerate tumor neovascularization in a VEGF-independent manner (Unterleuthner et al. 2020; Wan et al. 2021). In CRC, WNT2 was observed to be selectively increased in CAFs. Proteomic analysis of CAFs by mass spectrometry and cytokine microarray analysis indicated that WNT2 overexpression elevated the expressions of angiogenic factors (IL-6, G-CSF, and PGF) (Unterleuthner et al. 2020). Therefore, tumor angiogenesis is closely related to the tumor-promoting effects of CAFs. However, some studies have also reported that certain CAF subsets produce the angiogenesis inhibitor THBS1 (Kalluri 2016; Palma et al. 2017). Recent application of single-cell technology has revealed that CAFs are comprised of heterogeneous cell subsets with different precursors and diverse functions in the TME (Lei et al. 2021). Therefore, the specific functional roles of distinct CAF sub-populations in promoting angiogenesis require further study.

### Anti-tumor functions of heterogeneous CAF subsets

Emerging evidence has suggested that CAFs act as promoters of tumor growth, invasion, metastasis, and angiogenesis. An in-depth understanding of tumor biology has revealed that CAFs are stromal cell types with heterogeneous functions. The application of newly developed single-cell sequencing technology has enabled the exploration of cell heterogeneities with single-cell resolution. It has also revealed anti-tumor CAF subsets (Kobayashi et al. 2019; Liu et al. 2019; Menezes et al. 2022).

CAFs exert their tumor-suppressive functions via the following mechanisms: They promote anti-tumor immune cell infiltration, activate tumor inhibitory signaling pathways, restore chemoradiotherapy sensitivity, and cause the secretion of certain ECM components with tumor inhibitory functions (Chen et al. 2021a, b, c). Higher levels of αSMA-positive CAFs are associated with better survival outcomes than depletion of αSMA-positive myofibroblasts in transgenic mice, which results in the formation of an immunosuppressive microenvironment mediated by CD4^+^ FOXP3^+^ Tregs (Ozdemir et al. 2014). A recent study on PDAC combined the use of single-cell sequencing technology with pancreatic cancer mouse models. The study findings demonstrated the existence of two CAF subsets (tumor-promoting FAP-positive CAFs and tumor-inhibitory αSMA-positive CAFs) with completely opposite functions in the TME (McAndrews et al. 2022). CAFs expressing αSMA can inhibit tumor growth by increasing the infiltration ratio of T effector cells (McAndrews et al. 2022). αSMA-positive CAFs in the tumor stroma exert anticancer effects by mainly enhancing the degree of anticancer immunity. The tumor-inhibitory αSMA-positive CAFs are driven by specific tumor-suppressive signaling pathways (Chen et al. 2021a, b, c; Lee et al. 2014; Shin et al. 2014; Tanaka et al. 2022), such as the SHH signaling pathway. This pathway mediates the tumor-inhibitory functions of CAFs via a stromal response in pancreatic (Lee et al. 2014) and bladder (Shin et al. 2014) cancers, thereby impeding cancer progression.

Tumor-inhibitory CAF subgroups can also interact with tumors by secreting various cytokines, chemokines, and proteins that restore chemotherapy sensitivity. In ER + breast cancer, there are two types of CAFs, which are defined by CD146 expression, that exhibit distinct responses to tamoxifen therapy (Brechbuhl et al. 2017). CD146-negative CAFs suppress estrogen receptor expression and increase tumor cell resistance to tamoxifen. In contrast, CD146-positive CAFs sustain tumor cell ER expression, maintain tamoxifen sensitivity, and improve overall survival rates (Brechbuhl et al. 2017). Similarly, CAFs can be classified into three subtypes based on HGF and FGF7 expressions in NSCLC cells. Subtype III CAFs (low HGF/low FGF7) are chemo-attractants that promote tumor-killing immune cell infiltration that reverses ICB tumor resistance (Hu et al. 2021a, b). In addition, some CAF types can inhibit tumor growth by targeting tumor-inhibitory signaling pathways (Maris et al. 2015; Mizutani et al. 2019; Zhang et al. 2022). Asporin, a newly discovered TGF-β1 inhibitor, is derived from breast cancer stromal fibroblasts and activated by hormone receptor positive cancer cells. However, no TNBC cells are expressed at low levels in normal breast tissue. Recombinant asporin and synthetic peptide fragments inhibit MDA-MB-468 cells by inhibiting TGF-β1-mediated SMAD2 phosphorylation and EMT, which significantly reduces tumor growth (Maris et al. 2015; Mizutani et al. 2019). CAFs also impair tumor invasion by ECM remodeling. Researchers established a conditional knockout mouse model of the host VCAN and observed that the loss of VCAN in mice promoted tumor cell proliferation, followed by angiogenesis. It also caused a reduction in CAF numbers and collagen fiber levels as well as dysregulation of ECM structure. However, the re-expression of VCAN in tumor cells results in its re-integration into the ECM. This leads to the restoration of collagen fiber levels and recovery of CAF numbers as well as heir tumor inhibition ability (Fanhchaksai et al. 2016). Hence, the discovery of the opposing functional phenotypes of CAFs-derived VCAN emphasizes its double-edged role in the development and progression of diverse cancer types (Yeung et al. 2013; Chida et al. 2016; Kato et al. 2022). TGF-β mediated up-regulation of VCAN expression in CAFs can facilitate the invasion potential of ovarian cancer cells through the NF-κB signaling pathway (Yeung et al. 2013). Stromal VCAN is also a poor prognostic factor in HCC and CRC (Chida et al. 2016; Kato et al. 2022). Therefore, the biological function of VCAN requires further elucidation. Further basic science research is required to investigate and identify the mechanisms through which it interacts with and exerts its effects on the heterogeneous TME. The mechanisms by which some CAF subsets express their tumor suppressive effects has been extensively explored previously. Hence, CAF-targeted treatments should focus only on CAFs that promote the development and progression of malignant tumors and not on the entire CAF subsets. Considering the origins and heterogeneity of CAFs, previous CAF markers could be widely expressed in other cell types. Therefore, it is difficult to establish a CAF-specific signature. Emerging single-cell sequencing technology can be used to depict the biological landscape of tumors. The widespread application of single-cell technology to investigate the TME could lead to the accurate identification of cancer-promoting and cancer-suppressing CAFs with special characteristics. This could provide a new perspective for the application of cancer-promoting CAF-based therapy. The biological characteristics of anti-cancer CAFs subsets have been elaborated in Table 1.

**Table 1:** Heterogeneous CAF subsets with anti-tumor functions

Cancer type	CAF subtype	Biological effect	Refs.
ER^+^ breast cancer	CD146^pos^ CAFs	Maintain ER expression and sustain sensitivity to tamoxifen	Brechbuhl et al. (2017)
Pancreatic cancer	α-SMA^+^ CAFs	Mediate T-effector cell to Treg ratio and promote immune infiltration	McAndrews et al. (2022)
Breast Cancer	Asporin^+^ CAFs	Inhibition of TGF-β1-mediated SMAD2 phosphorylation, EMT process and stemness	Maris et al. (2015)
Fibrosarcoma	VCAN^+^ CAFs	Remodel the ECM and maintain the tumor-inhibitory functions	Fanhchaksai et al. (2016)
Lung cancer	low-HGF/low-FGF7 CAFs	Exert chemoattraction for immune cell infiltration and reverse ICB resistance	Hu et al. (2021a, b)
Pancreatic cancer	Meflin^+^ CAFs	Suppress the progression of tumor	Mizutani et al. (2019)
Pancreatic cancer	α-SMA^+^ CAFs	Inhibit accumulation of CD4 + FOXP3 + Tregs and immunosuppressive microenvironment formation	Ozdemir et al. (2014)

### CAF participation in cell-to-cell communication within the TME

**Fig. 2:**
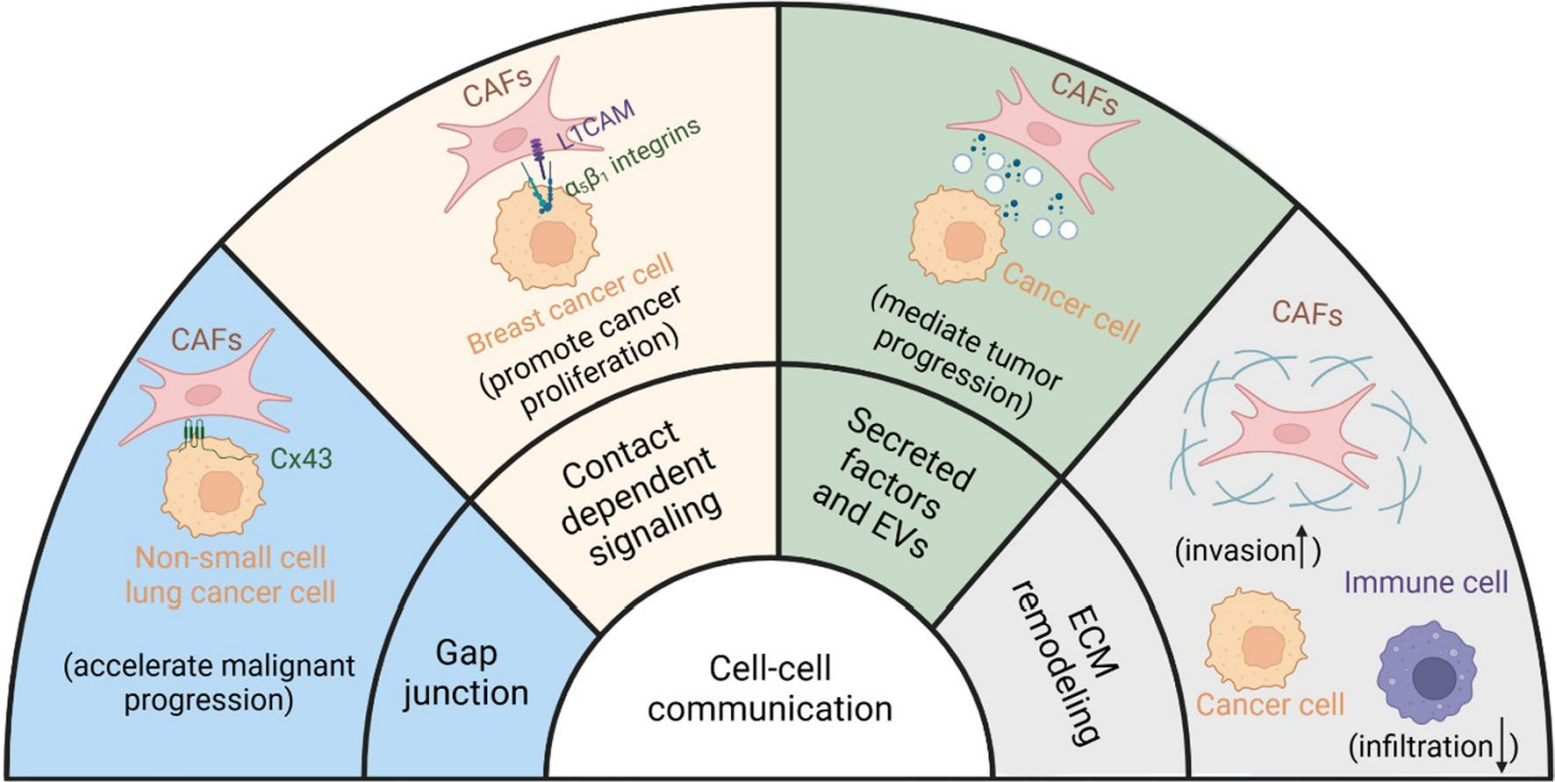
Examples illustrate the cell-to-cell communications CAFs participated. To reshape the TME, CAFs could interact with other cells by direct cell-cell contacts or indirect mechanisms, including: (1) gap junctions and (2) contact-dependent signaling both belong to direct cell junctions, (3) secreted soluble factors and EVs-mediated cell interactions, and (4) CAF-mediated ECM remodeling

Previous studies have reported that CAFs exert their tumor-promoting or -inhibitory effects through interactions with other cells and non-cellular TME components (Li et al. 2021). This interplay could occur via different mechanisms, such as direct cell-cell contact or indirect mechanisms, which include the remodeling of ECM components and secretion of various bioactive components (Li et al. 2021) (Fig. 2)

### Direct interactions through the cell junctions

CAFs can directly interact with other cells and non-cellular TME components through cell junctions. CAFs have gap junctions (Liu et al. 2022a, b; Luo et al. 2018; Maeda et al. 2009), tunnel nanotubes (Pinto et al. 2020; Roehlecke and Schmidt 2020), and the ability to perform contact-dependent signaling (Busch et al. 2015; Nakaoka et al. 2017). Many small molecules and metabolites can be delivered from CAFs to recipient cells via junctions. This helps mediate the corresponding recipient cell signaling pathway and control the downstream functional gene expression. In NSCLC, CAFs accelerate cancer cell proliferation, migration, and EMT through unidirectional gap junctional intercellular communication mediated through connexin 43 (Luo et al. 2018). NSCLC cells exhibit increased oxidative phosphorylation because of CAF stimulation via the abovementioned mechanism. This results in ATP production, which activates the PI3K/AKT and MAPK/ERK signaling pathways in cancer cells that are responsible for the maintenance of malignant phenotypes (Luo et al. 2018). In addition, CAFs communicate with other cells by binding to ligand receptor pairs in a cell-cell contact-dependent manner. This leads to changes in biological functions. Mint3 regulates L1CAM expression in fibroblasts. It activates the ERK signaling pathway in human breast cancer cells by binding to integrin α5β1. Hence, it is able to regulate CAF-tumor cell crosstalk (Nakaoka et al. 2017).

### Indirect contacts by ECM remodeling and secreted molecules

CAF-mediated ECM remodeling indirectly affects the communication between tumor cells and other interstitial cells. CAFs enhance tumor cell malignancy and promote invasion and metastases by remodeling ECM components. CAF-induced ECM stiffness effectively blocks the infiltration of tumor-killing immune cells (Mao et al. 2021; Calvo et al. 2013; Kaur et al. 2019; Monteran and Erez 2019). Various directly secreted soluble factors and bioactive molecules are transported through vesicles (such as non-coding RNAs, functional proteins, nucleic acids, and metabolites). These play essential roles in CAF-mediated indirect cell-cell communication (Kalluri and LeBleu 2020; Li et al. 2021; Peng et al. 2021a, b). Exosomes are originally endosome EVs. They measure approximately 40–160 nm in diameter and can transport signaling molecules to recipient cells (Kalluri and LeBleu 2020). Multiple studies have conducted in-depth exosome analyses in the TME. They have demonstrated functional roles of CDEs in tumor-associated cell-cell communications. Hence, the therapeutic targeting of CDEs or the transportation of anti-tumor drugs with exosomes might decrease these tumor-promoting interactions. This can have curative value. Therefore, it is important to understand the biological roles of CDEs in the TME. The following sections focus on the functions, mechanisms, and clinical significance of CDEs.

### Biological origins and characteristics of exosomes

EVs are a class of transport vesicles secreted by most cells that contain various bioactive molecules with cell-to-cell communication mediation abilities. The classification of EV subtypes is based on (a) physical characteristics such as size, ranges or density, (b) biochemical composition, and (c) descriptions of conditions or cell of origin (Thery et al. 2018). EVs could also divided into two major categories according to their size, content, and biological functions: ectosomes and exosomes (Kalluri and LeBleu 2020; Wiklander et al. 2015). Of which, ectosomes are vesicles with ranges in size from 50 nm to1µm which generated by direct outward budding of the plasma membrane, containing microvesicles, microparticles and so on. Exosomes are EVs with a diameter of 40–160 nm. They are mainly derived from multivesicular bodies (MVBs) and formed by plasma membrane invagination (Kalluri and LeBleu 2020).

**Fig. 3:**
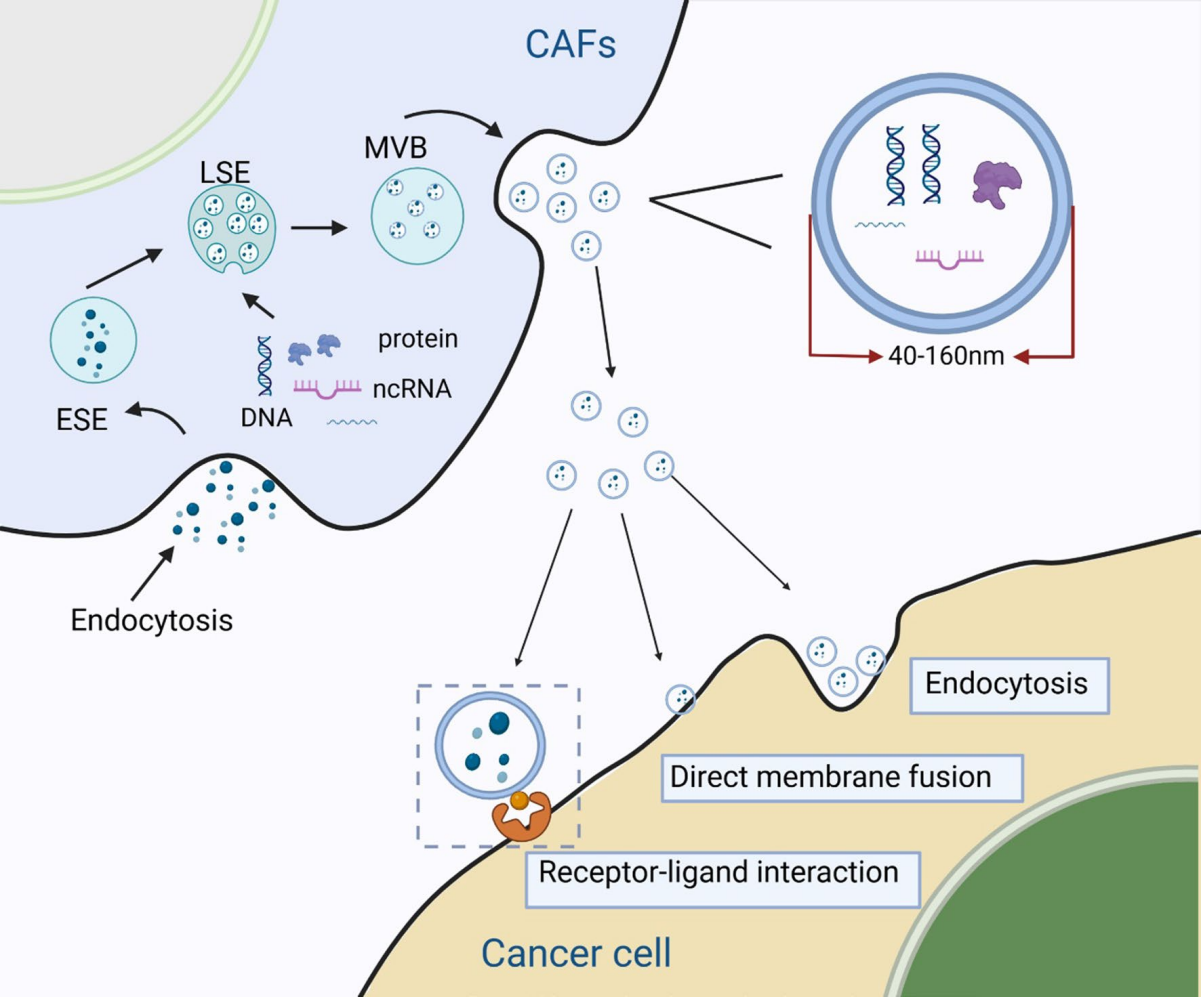
Synthesis of CDEs and its interactions with cancer cells. Exosomes are defined as EVs with a diameter of 40–160 nm carrying various biomolecules including nucleic acids, lipids, metabolites, and cell-surface proteins. Exosome formation depends on three important processes: endocytosis of various biomolecules and the formation of early sorting endosome (ESE) followed by late sorting endosome (LSE), and then synthesis of intracellular MVBs containing intraluminal vesicles (ILVs). Finally, MVBs fuse with the cell membrane, releasing exosome contents to the extracellular regions via exocytosis. Signal interaction patterns are mainly determined by exosome-transported molecules in three ways: direct cell membrane fusion with recipient cells, endocytosis, and internalization triggered by the binding of specific molecules on the exosome surface to corresponding receptor cells (receptor-ligand interaction)

According to original cell sources, exosomes carry various highly heterogenous biomolecules, including nucleic acids, lipids, metabolites, and cell-surface proteins (Pathan et al. 2019). Exosome formation depends on two important processes: double cell membrane invagination and the formation of intracellular MVBs containing intraluminal vesicles (ILVs). The cell membrane undergoes the first invagination and forms a cup-like structure that contains cell surface proteins, extracellular microenvironment-related proteins, and metabolites. This is followed by the formation of early sorting endosomes, which subsequently mature into late-sorting endosomes. Following this, the endosomal membranes undergo inward invagination to form MVBs containing ILVs (exosomal precursors). Finally, MVBs that are not degraded by lysosomes or autophagosomes fuse with the cell membrane. This results in the release of exosomal contents to the extracellular regions via exocytosis (Kalluri and LeBleu 2020; Kourembanas 2015). Exosomes were initially considered to be responsible for the removal of unwanted substances, such as proteins and lipids. However, it has recently been discovered that exosomes are also involved in the regulation of signal communication between cells (Huang-Doran et al. 2017; Tkach and Thery 2016). Signal communication patterns are mainly determined by exosome-transported molecules in three ways: cell membrane fusion with recipient cells, endocytosis, and internalization, which is triggered by the binding of specific molecules to corresponding receptors on the exosomal surface (Chen et al. 2021a, b, c; Mathieu et al. 2019) (Fig. 3)

In addition to their normal physiological processes, exosomes are also involved in signal transduction during the progression of diseases such as cancer. Information is transmitted among stromal, immune, and tumor cells by the exchange of signals through exosomes. Thus, exosomes regulate the activity of signaling pathways, which promotes tumor progression and immune-related inflammation (Kalluri and LeBleu 2020; Zhang and Yu 2019). Hence, CDEs are involved in shaping the TME as they participate in signal crosstalk among CAFs, tumor cells, and immune cells (Li et al. 2021; Zhang and Yu 2019).

**Table 2:** CDE biomolecules in the TME

Cancer type	Biomolecule	Mechanism	Biological effect	Refs.
Gastric cancer	miRNA-522	Inhibition of cancer cells ferroptosis by targeting ALOX15 and lipid-ROS accumulation	Decreased chemo-sensitivity	Zhang et al. ( 2020a, b, c)
Head and neck cancer	miRNA-196a	Bind to CDKN1B and ING5	Acquire cisplatin resistance	Qin et al. (2019)
Colorectal cancer	lncRNA H19	Active β-catenin pathway by sponging tumor-inhibitory miR-141	Promote tumor stemness and drug resistance	Ren et al. (2018a, b)
Colorectal cancer	circSLC7A6	Mediate miRNAs to regulate the pro-tumor effect of CXCL5	Promote tumorigenesis	Gu et al. (2020)
Hepatocellular cancer	Gremlin-1	By regulating Wnt/β-catenin and BMP signaling	Promote tumor progression and decrease the sensitivity to sorafenib	Qin et al. (2022)
Pancreatic cancer	miRNA-320a	By mediating PTEN/PI3Kγ signaling	Facilitate the M2 polarization of TAMs	Zhao et al. (2022)
Breast cancer	miRNA-92	Increase PD-L1 expression to impair the functions of T cells	Suppress the functions of anti-tumor immune cells	Dou et al. (2020)
Lung cancer	THBS2	Inhibit T cell proliferation	Decrease immune cells infiltrations	Yang et al. (2022)
Esophageal squamous cell cancer	miRNA-21	IL-6 and miRNA-21 activate the STAT3 signaling to induce M-MDSCs generation	Promote tumor resistance to cisplatin	Zhao et al. (2021)
Colorectal cancer	LINC00659	Directly interact with miRNA342-3p to increase ANXA2 expression in cancer cells	Promote tumor progression	Zhou et al. (2021)
Breast cancer	circHIF1A	Sponge miRNA-580-5p to modulate the expression of CD44	Promote tumor cell stemness	Zhan et al. (2021)
Ovarian cancer	TGFβ1	Activate the SMAD signaling pathway	Promote EMT process	Li et al. (2017)
Cervical cancer	miRNA-1323	Target PABPN1 to regulate Wnt/GSK-3β/β-catenin pathway	Promote tumor progression and radio-resistance	Fang et al. (2022)
Prostate cancer	miRNA-1290	Inhibit GSK3β/β-catenin signaling pathway	Facilitate tumor growth and metastasis	Wang et al. (2022a, b)
Oral squamous cell cancer	miRNA-34a-5p	Reduced miR-34a-5p up-regulate AXL and activate AKT/GSK-3β/β-catenin signaling pathway	Promote EMT process and cancer metastasis	Li et al. (2018)
Gastric cancer	circ_0088330	Sponge miR-1305 to regulate JAK/STAT signaling pathway	Promote tumor progression	Shi et al. (2021)
Colorectal cancer	circN4BP2L2	Up-regulate ELF4A3 to regulate PI3K/AKT/mTOR axis	Promote CRC chemoresistance and stemness	Qu et al. (2022)
Bladder cancer	miRNA-148b-3p	Target PTEN and activate Wnt/β-catenin signaling pathway	Promote EMT process and metastasis	Shan et al. (2021)
Laryngeal cancer	miRNA-34c-5p	Loss of miR-34c-5p promote the cancer cells malignant phenotypes	Promotion of stem-like phenotypes of cancer cells	Wang et al. (2022a, b)
Breast cancer	SNHG3	Sponge miR-330-5p to regulate the expression of PKM and inhibit mitochondrial oxidative phosphorylation and increase glycolysis carboxylation	Promote tumor progression	Li et al. (2020)

### CDE biomolecules in the TME

Considering distinct cell sources, heterogeneous cellular microenvironments, and different cell states, the corresponding exosomes contain different bioactive molecules that perform diverse functions (Kalluri and LeBleu 2020). Recently, the functional roles of various signaling molecules contained in CDEs in the TME have been highlighted. The various molecular mechanisms mediated by CAF-derived exosomes have also been extensively explored (Peng et al. 2021a, b). Cancer cells and other cells ingest different molecules contained in CDEs. Through this, they regulate their own downstream signaling pathways and the levels of numerous signaling factors, including nucleic acids, functional proteins, and small metabolic molecules (Li et al. 2021; Peng et al. 2021a, b; Yang et al. 2017). The details have been presented in Table 2.

#### Non-coding RNA

**Fig. 4:**
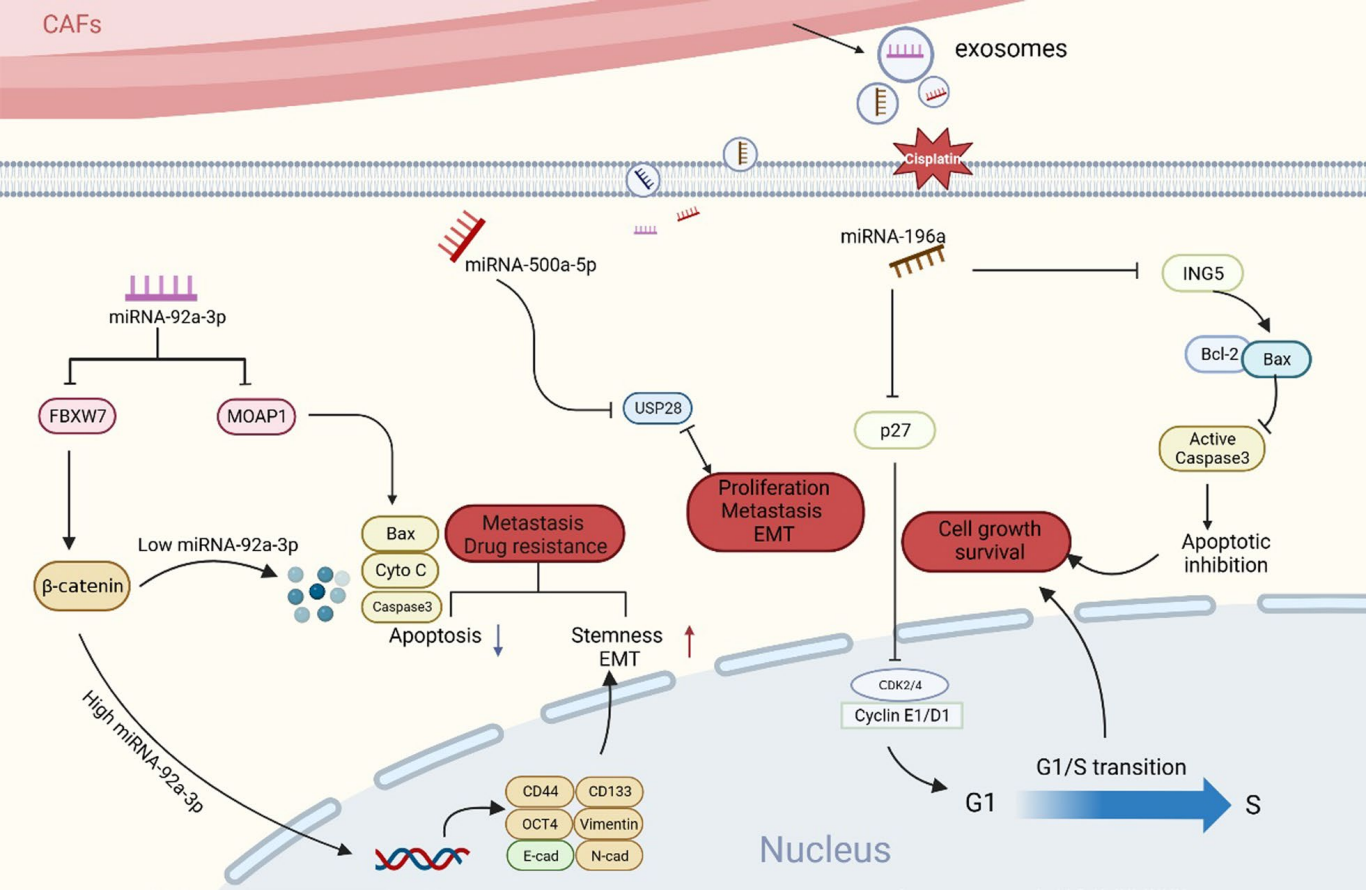
Examples illustrate the mechanisms of CDEs contained non-coding RNA (miRNA) interact with other cells. Exosome-packed miRNA-92a-3p secreted by CAFs directly suppress the expression of FBXW7 and MOAP1 in CRC cells, which contribute to tumor stemness, metastasis, EMT and therapy resistance (Hu et al. 2019a, b). CDEs derived miRNA-500a-5p prompt breast cancer malignant behaviors (proliferation and metastasis) by directly targeting USP28 (Chen et al. 2021a, b, c). Exosomal miRNA-196a derived from CAFs targeting CDKN1B and ING5 confer head and neck cancer cells cisplatin resistance (Qin et al. 2019)

CDE-packed non-coding RNAs have recently emerged as a prominent topic. Among these, miRNAs are the main research targets. Their functional mechanisms in the TME have been widely investigated. Moreover, miRNAs are endogenous single-stranded nucleic acid sequences with a length of 20–24 bases. They target the 3’-untranslated region of mRNAs which is matched with their specific sequences. They negatively regulate their expression, thereby affecting downstream signaling transduction pathways (Bartel 2004; Saliminejad et al. 2019). In the TME, CDEs containing various miRNAs are internalized by recipient cells. This causes a reduction in mRNA expression as well as various phenotypic changes such as tumor progression, therapy resistance, metastasis, and metabolic reprogramming (Zhang et al. 2020a, b, c; Peng et al. 2021a, b; Qin et al. 2019; Fang et al. 2022; Wang et al. 2022a, b). Similarly, hnRNPA1 regulates the packaging of miR-196a into CDEs. When taken up by tumor cells, miR-196a could inhibits CDKN1B and ING5, endowing advanced HNC cells with resistance to cisplatin. The depletion of exosome or exosomal miR-196a from CAFs results in drug sensitivity restoration in HNC cells. This highlights the potential value of CDEs-derived miR-196a as therapeutic targets in HNC (Qin et al. 2019) (Fig. 4).

Long non-coding RNA (lncRNA) has a length > 200 nucleotides with no protein-coding ability (Peng et al. 2021a, b; Schmitt and Chang 2016). Furthermore, lncRNAs modulate gene expression in transcriptional, post-transcriptional, and epigenetic regulation by interacting with DNAs, RNAs, and proteins (Statello et al. 2021). In addition, the localizations of different lncRNA in the nucleus or cytoplasm lead to distinct functions. Cytoplasmic lncRNAs mainly exert tumor-promoting functions by absorbing miRNAs or binding functional proteins. In contrast, nuclear lncRNAs bind transcription factor-binding proteins and regulate the transcriptional activity of cancer-related genes (Peng et al. 2021a, b; Statello et al. 2021). Exosomal lncRNA entry into tumor cells regulates their mRNA expression via one of the aforementioned molecular mechanisms (Ren et al. 2018a, b; Zhou et al. 2021; Deng et al. 2020). In CRC, lncRNA H19 (Ren et al. 2018a, b) and LINC00659 (Zhou et al. 2021) in CDEs act as sponges that competitively absorb endogenous microRNAs with tumor suppressive functions. This results in their inability to inhibit tumors and indirectly activate pro-tumor signaling pathways to promote tumor progression. Exosome-derived circRNAs are also involved in tumorigenesis (Gu et al. 2020; Zhan et al. 2021; Chen and Shan 2021). Exosomal circSLC7A6 secreted by CAFs regulates CRC cell proliferation and invasion and inhibits cell apoptosis by targeting the chemokine receptor CXCR5 (Gu et al. 2020). Therefore, the application of inhibitors targeting pro-tumor exosomal non-coding RNA (ncRNAs) provides a new theoretical basis for clinical treatment targeting CDEs.

#### Functional proteins

Proteins are responsible for all the cellular functions in the body, including both physiological and pathological functions. Similarly, in the TME, various proteins contained in exosomes play major regulatory roles in communication with other cells. Exosomes contain different proteins in different microenvironments. These proteins interact, via complex mechanisms, with tumor cells, immune cells, and the ECM to maintain tumor phenotypes. Proteins contained in CDEs are released via paracrine or telecrine signaling. They are absorbed by recipient cells, and intercellular communications occur by the activation or inhibition of receptor cell signaling pathways. Some common cancer-related signaling pathways, including the TGF-β and WNT signaling axes, have been extensively investigated in CDE-related research (Qin et al. 2022; Chandra Jena et al. 2021). After secretion by CAFs, exosome-derived Gremlin-1 regulates the classical WNT and BMP signaling pathways in cancer cells. It also contributes to tumor progression (Qin et al. 2022). Similar mechanisms involving the abovementioned classic pathways have also been observed in other tumor types, including breast and ovarian cancers (Li et al. 2017; Luga et al. 2012). Hence, there is a possibility for tumor growth prevention through the delivery of drugs that target these common pathways. These targeted drugs could block these processes and reduce reciprocal support between cancer cells and stromal components.

### CDE maintenance of tumor cell malignancy

#### Proliferation, invasion and angiogenesis

CDEs are involved in the maintenance of tumor-related malignant phenotypes as they deliver various biological macromolecules and chemicals (such as proteins, ncRNA, mRNAs, and metabolites) that mediate local and systematic cell-to-cell communication in the TME (Li et al. 2021; Yang et al. 2019).

ncRNAs are important in the study of exosomes. They are molecular signal regulators of recipient cells and promote tumor progression in most cases (Yang et al. 2016). miRNAs are the most commonly reported bioactive molecules contained in exosomes. Numerous studies have revealed that CAF-derived exosomal-packed miRNAs are closely associated with cancer progression (Li et al. 2018; (Chen et al. 2021a, b, c; Wang et al. 2020; Yin et al. 2021; Dai et al. 2022). miR-34a-5p, which is derived from normal fibroblasts in oral squamous cell carcinoma (OSCC), inhibits OSCC cell proliferation and metastasis by binding to the target AXL. However, miR-34a-5p expression in CDEs is significantly reduced. This prevents the inhibition of the EMT process activated by the AKT/GSK-3β/β-catenin signaling axis, hence, indirectly promoting OSCC cell growth (Li et al. 2018; Chen et al. 2021a, b, c). In CRC, miR-135b-5p, which are derived from CDEs, accelerate the angiogenesis, proliferation and migration of human umbilical vein endothelial cells by separately suppressing TXNIP (Yin et al. 2021) and FOXO1 (Dai et al. 2022). This has been observed in both in vitro and vivo experiments. Another study has reported that CDEs release miR-181d-5p, which promotes its binding to the transcription factor CDX2. This inhibits the expression of HOXA2 (a tumor suppressor) and promotes breast cancer tumorigenesis (Li et al. 2020). Furthermore, some circRNAs and lncRNAs may assist in tumor growth. Shi et al. (2021). In breast cancer, CAF-secreted exosomal lncRNA SNHG3 also acts as a molecular sponge for miR-330-5p. It also regulates pyruvate kinase expression and inhibits mitochondrial oxidative phosphorylation in addition to increasing glycolytic carboxylation and other metabolic processes, and promoting cancer cell proliferation (Li et al. 2020). In addition to ncRNAs, different functional proteins contained in CDEs also influence tumor progression (Qin et al. 2022; Li et al. 2017; Chandra Jena et al. 2021; Hu et al. 2019a, b). In chemo-resistant CRC patients, CAFs secreted exosomal-VEGFA regulates angiogenesis and the development of cisplatin resistance phenotypes (Shi et al. 2022a, b). Other functional proteins such as TGF-β1 and Gremlin-1, which are also packed in CDEs, activate different tumor-promoting signaling pathways and enhance the tumor aggressive phenotype in ovarian cancer and HCC (Qin et al. 2022; Li et al. 2017).

#### Treatment tolerance

Treatment tolerance is an important cause of tumor recurrence. Multiple recent studies have confirmed that exosomes originating from benign or malignant cells in the TME also regulate treatment tolerance (Khalaf et al. 2021; Milane et al. 2015; Quail and Joyce 2013). Cell-cell signal communication between CDEs, tumor cells, and immune cells is a potential mechanism for treatment tolerance (Erin et al. 2020). During the development of drug resistance, CAFs release exosomes, including proteins and miRNAs (Qin et al. 2019; Shan et al. 2020, 2021; Gao et al. 2020; Richards et al. 2017), which are endocytosed by cancer or immune cells. They subsequently bind to various downstream molecular targets, thereby altering signaling pathways and conferring anti-tumor drug tolerance in tumor or immune cells. In HNC research, it was observed that CAFs, which are intrinsically resistant to cisplatin, deliver functional miR-196a to tumor cells through exosomes. Hence, they endow cisplatin resistance by targeting CDKN18 and ING5 in HNC cells (Qin et al. 2019). In contrast, CDEs reduce cancer cell sensitivity to drug therapy via ECM remodeling and promotion, as well as tumor metastasis (Qin et al. 2022; Shan et al. 2021; Shibue and Weinberg 2017). In a bladder cancer xenograft mouse model, Shan et al. observed that CDEs increased miR-148b-3p expression, with PTEN (with tumor-inhibitory functions) confirmed as its target. It promoted EMT and cancer cell metastasis by activating the WNT/β-catenin signaling pathway and reducing cancer cell chemosensitivity (Shan et al. 2021). Hence, our in-depth discussion of various drug resistance mechanisms mediated by CDEs suggests that appropriate methods should be developed to impede the secretion and release of these exosome-secreted molecules. This will effectively inhibit CAF-induced therapeutic tolerance, thereby alleviating or halting anticancer drug tolerance to a certain extent.

#### Metabolic reprogramming

Tumor metabolic reprogramming is a hallmark of cancer. Due to the presence of contradictory microenvironment factors such as limited nutrients and the need for various nutrient substances among cancer and stromal cells, many cells in the TME undergo reprogramming of metabolic patterns and extensive metabolic crosstalk to maintain tumor metabolic homoeostasis (Bader et al. 2020). CAFs are the most common cell type in the tumor stroma. They trigger tumor metabolic reprogramming during the stimulation of TME components (Martinez-Reyes and Chandel 2021). CDEs are taken up by breast cancer cells, resulting in promotion of glycolysis and inhibition of oxidative phosphorylation inhibition (Zhang et al. 2020a, b, c). In human prostate and pancreatic cancer cells, CDEs inhibit mitochondrial oxidative phosphorylation and increase glycolysis and glutamine-dependent reductive carboxylation (Zhao et al. 2016). CDEs also transport essential amino acids, lipids, and other metabolic components of CAFs, thus maintaining tumor growth (Zhao et al. 2016). However, although many studies have reported a close relationship between CAFs and tumor metabolic reprogramming, little is known about the specific mechanism by which CDEs intervene in this process. Hence, studies focusing on CDE-mediated tumor metabolism reprogramming are required. Theoretically, the various bioactive molecules carried by CDEs are important tumor metabolism regulators. They can be helpful in the development of therapeutic strategies targeting drug resistance and metastasis caused by metabolic changes in cancers.

##### CDE-induced cancer stem cell (CSC) formation

Continuous self-renewal and the sustenance of tumor proliferative capabilities are considered a hallmark of cancer (Hanahan 2022). The tumor phenotype is mainly attributed to a small number of tumor cell populations with self-renewal ability, known as CSCs. These are important factors in cancer progression, metastasis, and recurrence (Chen et al. 2021a, b, c). In the last decade, many CSCs have been identified. They have diverse marker genes in different tumor types. The depletion of these cells may significantly decrease tumor cell self-renewal and proliferative ability. This indicates that CSCs may be the optimal tumor treatment target (Al-Hajj et al. 2003; Batlle and Clevers 2017; Ricci-Vitiani et al. 2007).

In recent years, it has been demonstrated that inherent genetic or epigenetic alterations and external signal communication between distinct cells in the TME shape CSC heterogeneity. This affects the tumoral process and drug sensibility, thereby considerably restricting CSC-targeted therapeutic outcomes (Prasetyanti and Medema 2017; Hu et al. 2019a, b). Our understanding of tumorigenic mechanisms has shifted from a tumor cell-centered viewpoint to the TME components co-mediated perspective. This has broadened our understanding of biological characteristics of tumors. Proteins, nucleic acids, exosomes, and other signaling molecules that are secreted from CAFs play several roles in transferring signals and promoting tumor cell stemness (Alguacil-Nunez et al. 2018). Exosomes are endosome-derived EVs that contain numerous signaling molecules, especially various non-coding RNAs. These RNAs play an important role in CAF-induced tumor cell stemness (Zhan et al. 2021; Qu et al. 2022; Hu et al. 2015; Lin et al. 2019; Liu et al. 2020; (Wang et al. 2022a, b). circHIF1A (Zhan et al. 2021) and cricN4BP2L2 (Qu et al. 2022) contained in CDEs also regulate pro-tumor downstream signaling pathways by acting on tumor cells, thereby facilitating therapy resistance and tumor cell stemness. Furthermore, the reduction in tumor-suppressive signaling molecules in CDEs indirectly enhances the development of cancer. The expression of miR-34c-5p with its tumor-inhibitory function decreases in CDEs, which indirectly promotes proliferation, invasion, drug resistance, and the maintenance of stem-like phenotype in laryngeal cancer cells (Wang et al. 2022a, b).

The interaction between CDEs and CSCs has been studied in different tumors. The targeting of signal communication between these cells is theoretically the best way to reduce stem cell formation and tumor recurrence. CAFs are cell subsets with different functions, and CSCs also exhibit significant heterogeneity. Hence, the abovementioned mutual interaction is complicated, and it remains unclear whether tumor-suppressive CAFs are involved in reducing tumor stemness. However, it may be counterproductive to inhibit CAF activity without considering other cell subgroups that possess tumor inhibitory potentials.

### Crosstalk between CDEs and immune cells

The primary role of CDEs in the TME is tumor growth, metastasis, and promotion of drug resistance through tumor cell endocytosis and the activation of downstream carcinogenic signaling pathways (Li et al. 2021). Many non-tumor cells that exist simultaneously in the TME are also important recipient cells for CDEs. Immune cells are the main non-tumor cell type. Emerging evidence has demonstrated that there are extensive interactions between CAFs and various types of immune cells, such as macrophages, T cells, Treg cells, and NK cells (Kalluri and LeBleu 2020; Li et al. 2021; Mao et al. 2021). CDEs bridge the cell-cell communication between CAFs and immune cells to shape the immunosuppressive microenvironment. However, the mechanisms by which CDEs regulate tumor immunity remain largely unknown. Therefore, further research investigating these mechanisms is warranted. In this study, we have focused on the interactions between CDEs and the different types of immune cells in the TME.

### CDEs-mediated macrophage differentiation

Monocytes are recruited into tumor regions under different polarizing chemokine and cytokine stimulations. They are subsequently polarized into two types of TAMs with opposite functions: M1-TAMs (tumor-suppression) and M2-TAMs (tumor-promotion) (Mantovani et al. 2004; Boutilier and Elsawa 2021). Some tumor-derived cytokines drive the polarization of macrophages into M2-TAMs. These induce angiogenesis, immunosuppression, and cancer cell metastasis. In addition, microenvironmental stimuli such as immune-related inflammation and hypoxia can trigger the transformation of M1-TAMs into M2-TAMs, which further impairs infiltrating immune cell function (Boutilier and Elsawa 2021; Zhang et al. 2019). CDEs produce an immunosuppressive microenvironment by promoting M2-TAM polarization in tumors (Su et al. 2021). CDE-derived miRNA-320a is internalized by macrophages. It induces M2-TAM polarization through the PTEN/PI3Kγ signaling pathway, thereby enhancing pancreatic cancer malignancy (Zhao et al. 2022).

### The interaction of CDEs and other immune cells

In addition to the formation of an inhibitory microenvironment by macrophage polarization, CDEs interact with other immune cells, including T cells and monocytic myeloid-derived suppressor cells (M-MDSCs). CDEs also impair the function and infiltration capacity of effector immune cells, thereby producing immunosuppressive and chemotherapy-resistant phenotypes (Dou et al. 2020; Yang et al. 2022; Zhao et al. 2021). Exosome-derived thrombospondin 2 (THBS2) inhibits T cell proliferation. Hence, lung adenocarcinoma with high THBS2 expression demonstrates less immune infiltration and more T cell depletion (Yang et al. 2022). In addition to immune effector cell function impairment, CDEs exert their effects on immunosuppression and drug resistance by inducing monocyte differentiation into immunosuppressive M-MDSCs via the following specific mechanism: CDE-derived miR-21 reduces PTEN expression in monocytes; phosphorylated NF-κB promotes downstream IL-6 expression; and CAF-secreted IL-6 acts on monocytes in a paracrine manner. IL-6, released through paracrine and autocrine mechanisms activates the STAT3 signaling pathway of monocytes. It also promotes their differentiation into M-MDSCs, thereby inducing immunosuppression and cisplatin resistance (Zhao et al. 2021).

Although studies have demonstrated mutual interactions between CAFs and immune cells, many internal mechanisms have not yet been clearly explained due to the complexity of the TME. The abovementioned study indicated that blocking the crosstalk between CAFs and immune cells can theoretically alleviate the immunosuppressive microenvironment and reverse ICB resistance.

### Clinical perspectives of CDEs

#### Diagnostic applications of CDEs

**Fig. 5:**
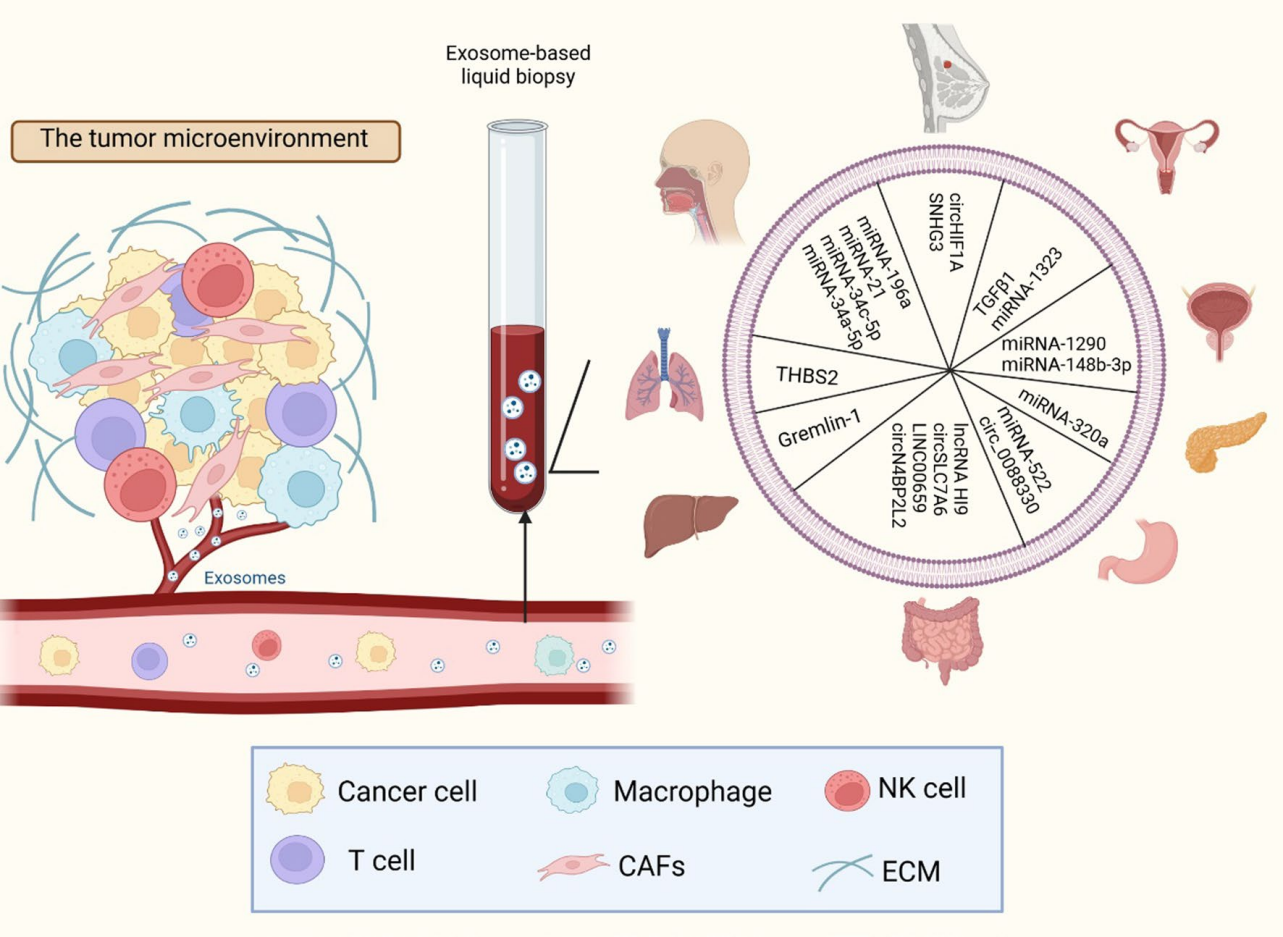
CDEs-based liquid biopsy strategies for cancer diagnostic applications. Considered the facts that CAFs-derived exosomal-packed biomolecules fully reflect the current tumor status, CDEs-based liquid biopsy are optimal minimally invasive detection indicators for cancer diagnosis, therapeutic efficacy assessment, and prognosis evaluation. By collecting the CDEs and detecting contained bioactive molecules from blood or other body fluids, we could not only enhance the patient compliance, but also improve the detectable rate combined with other detection indicators for early diagnosis

The pathological mechanisms of exosomes in diseases have been widely studied. Exosomes can be secreted by almost all cells. They transport the biomolecules delivering signals between cells and can be detected in all body fluids. Hence, the diagnostic applications of exosomes in various diseases, including cardiovascular, neurological, oncological, and hepatic diseases is being increasingly explored (Li et al. 2021; Kanninen et al. 2016; Liu et al. 2021; Masyuk et al. 2013; Zhang et al. 2017). The application of exosomes in cancer diagnosis has attracted the greatest attention in research (Liu et al. 2022a, b; Paskeh et al. 2022). Exosomal-packed signals reflect source cell functional status and tumor progression. Therefore, exosomes are minimally invasive detection indicators that could be used for early tumor diagnosis, treatment efficacy detection, and prognosis evaluation (Liu et al. 2022a, b) (Fig. 5).

Similarly, substances contained in CDEs also indicate tumor status and progress. They can provide valuable diagnostic information. Hence, such biomolecules can have a potential application in tumor diagnosis (Yin et al. 2021; Jiang et al. 2021; Kunou et al. 2021). Various ncRNAs that are differentially expressed in CDEs could be major candidates for tumor metastasis and therapy sensitivity assessment (Shi et al. 2022a, b; Fang et al. 2022; Wang et al. 2022a, b; Yin et al. 2021; Jiang et al. 2021; Kunou et al. 2021). For example, miRNAs such as miR-1290, miR-1323, miR-20a, miR-4717-5p and miR-135b-5p are signaling molecules contained in exosomes. They regulate a series of tumor-related phenotypes by targeting certain recipient cells in different tumor types, including CRC (Yin et al. 2021), prostate cancer (Wang et al. 2022a, b), NSCLC (Shi et al. 2022a, b), cervical cancer (Fang et al. 2022), and malignant lymphoma (Kunou et al. 2021). As these miRNAs are associated with the malignancy potential of tumors, the expression of these markers in CDEs can be used to objectively evaluate cancer status and the possibility of drug resistance (Kalluri and LeBleu 2020; (Shi et al. 2022a, b; Fang et al. 2022; Kunou et al. 2021; Richards et al. 2022).

CDE-derived functional proteins also have a diagnostic value (Qin et al. 2022; Chen et al. 2017; Ganig et al. 2021). Ganig et al. conducted a proteomic analysis of exosomes derived from CAFs and normal fibroblasts (NFs) in 13 patients with CRC. They observed that QSOX1 expression in NFs was decreased in the serum of patients with CRC (Ganig et al. 2021). Furthermore, they reported significantly lower QSOX1 expressions in patients with tumors compared with those in the control group (another independent cohort). This indicates that QSOX1 is a reliable non-invasive indicator for early tumor diagnosis. Gremlin-1, another functional protein with diagnostic potential, can also be released by CAFs into HCC cells in an exosome-packaged paracrine manner (Qin et al. 2022).

Exosomes exhibit considerable heterogeneity. Hence, their nucleic acids, proteins, lipids, and other components change based on the host cell type. Similarly, CDEs differ based on distinct tumor types and pathological states of the same tumor (Kalluri and LeBleu 2020; Zhu et al. 2020a, b). Therefore, the diagnostic applications of a single signature are limited. A combined analysis of multiple markers in exosomes is the optimal strategy for improving diagnostic sensitivity and specificity (Kalluri and LeBleu 2020; Liu et al. 2021; Zhu et al. 2020a, b). Presently, research teams have developed in situ detection technologies that can enable a simultaneous multiplexed detection of exosomal miRNAs and surface protein markers from prostate cancer exosomes in a single reaction. Therefore, this could provide good technical support for a joint diagnosis of exosome-based marker molecules in the future (Cho et al. 2019).

#### Exosomes-based therapeutical strategies

Exosomes are important transport vesicles for intercellular communication that carry nucleic acids, lipids, and proteins. Numerous tumor signaling molecules are endocytosed by recipient cells. This is followed by the activation of a series of biological processes, including tumor-driver gene expression mediation and immune cell infiltration regulation (Kalluri and LeBleu 2020). Exosomes can target specific receptor cells and regulate intercellular communication patterns. This makes it possible to apply exosome-targeted therapeutic regimens in oncotherapy (Zhu et al. 2020a, b; (Zhang et al. 2020a, b, c). However, the application of CDEs in cancer therapy has not been extensively studied. Therefore, we have mainly discussed exosomes-based treatment in this section. Therapeutic strategies for inhibiting the biosynthesis and release of exosomes from original cells as well as blocking exosome endocytosis by recipient cells have been widely investigated in many tumor types (Zhu et al. 2020a, b). Molecules, such as RAB27A (Johnson et al. 2016; Li et al. 2013; Martin et al. 2007), plectin (Li et al. 2013), and neutral sphingomyelinase (Trajkovic et al. 2008; Verderio et al. 2018) are closely related to exosome synthesis and secretion. Hence, they have been applied for targeted drug development. An increasing number of studies have confirmed that inhibition of these molecules can significantly suppress the tumor growth-promoting potential through pro-tumor exosome inhibition (Johnson et al. 2016; Datta et al. 2018; Lallemand et al. 2018). The exosome inhibitory effect of some drugs, such as tipifarnib (Martin et al. 2007; Colombo et al. 2014) and manumycin A (Datta et al. 2017) could selectively target tumor cell-released exosomes but exert no effects on normal cells. These pharmacological properties could reduce potential drug toxicity and allow for the more widespread clinical application of exosome-targeted therapy. Some compounds, including heparin, cytochalasin D, methyl-β-cyclodextrin, and dynasore can also potentially inhibit exosome uptake by target cancer cells. Therefore, they can restrain the activation of exosome-mediated tumor-promoting signaling pathways (Zhao et al. 2016; Bastos et al. 2018; Franzen et al. 2014; Kawamoto et al. 2012). The common compounds which could have applications as exosome-based therapies have been described in Table 3.

Exosomes are EVs secreted by body cells. Hence, they exhibit characteristics of bio-histocompatibility, stability, and low cytotoxicity. Moreover, exosomes target specific cells, which makes them ideal vehicles for anti-tumor drug delivery (Zhu et al. 2020a, b; Zhao et al. 2020). Methods of loading biomacromolecules or drugs into exosomes can be divided into two categories: direct carriers that transfer drugs into exosomes via liposomes or electroporation and indirect carriers that involve a co-culture of donor cells with drugs (Liu et al. 2021; Zhu et al. 2020a, b). Exosomes are transport vesicles that can be loaded with several biologically active molecules, such as functional proteins, nucleic acids, and chemical drugs (Liu et al. 2021; Zhao et al. 2020). miRNAs are the main signal regulatory molecules that are transported by exosomes. Distinct miRNAs are involved in tumor promotion and suppression. Loading miRNAs with tumor suppressive functions into exosomes of special donor cells and transporting them to tumor sites is an optimal anti-tumor strategy. Some studies have used MSCs as donor cells for exosomes. Bioengineered MSCs secrete exosomes containing miR-145-5p (Ding et al. 2019), miR-146b (Katakowski et al. 2013), and miR-379 (O’Brien et al. 2018) with tumor-inhibitory functions. These inhibit the malignant phenotypes of PDAC, breast cancer, and glioma cells. Kirsten rat sarcoma (KRAS) mutations are key drivers of pancreatic cancer. However, directly targeting this oncogene remains challenging. Kamerkar et al. (2017). This further substantiates the great potential of exosome-delivered therapy. Hu et al. also developed engineered exosomes vaccine against the FAP + CAFs and tumor cells by inducing cytotoxic T lymphocytes (CTLs), and promoting cancer cell ferroptosis by releasing IFN-γ from CTLs (Hu et al. 2021a, b). Other molecules such as proteins and chemotherapy drugs can also be transported to tumor cells after exosome engineering (Gilligan and Dwyer 2017).

Exosomes can be secreted by almost all cells. They act differently according to distinct donor cells. Thus, multiple studies have suggested that the pivotal roles of exosomes in regulating the tumor immune microenvironment can be ascribed to distinct immune cells. Therefore, engineering anti-tumor immune cells to regulate the synthesis and secretion of exosomes with immune activation functions is also an important topic in current research (Xu et al. 2020). The preparation of bioengineered immunoregulatory exosomes that could overcome the tumor immunosuppressive microenvironment and subsequently transport immune molecules into tumors to restore the level of immune infiltration has great prospects for clinical application.

**Table 3:** Compounds contained in exosome-based therapeutic strategies

Compound	Structure	Target	Refs.
Heparin		Inhibit the uptake of exosome by cancer cells via targeting cell-surface heparan sulphate proteoglycans	Christianson et al. (2013)
Cytochalasin D		Inhibit the actin polymerization and suppress the endocytosis of exosomes by cancer cells	Zhao et al. (2016)
Methyl-β-cyclodextrin		Remove cholesterol from membranes and interfere lipid rafts stability to inhibit the uptake of exosomes	Escrevente et al. (2011)
Dynasore		Inhibit the exosomes endocytosis mediated by dynamin	Kawamoto et al. (2012)
Gefitinib		Involve in a non-classical endocytosis pathway including dynamin and lipid-rafts	Hazan-Halevy et al. (2015)
Tipifarnib		Target RAB27A-participated exosome production or secretion process	Martin et al. (2007)
Manumycin A (MA)		Inhibit neutral sphingomyelinase (nSMase) thus reduce ceramide-regulated exosome release	Datta et al. (2017)

## Conclusions and future perspectives

CAFs are the primary stromal cells in the TME. Their biological characteristics have been extensively studied over the past decade. Extensive signal communications among CAFs, tumor cells, immune cells, and other interstitial cells helps induce and maintain the formation of various malignant biological phenotypes in tumor tissues. An increasing number of studies have demonstrated that targeting CAFs with tumor-promoting potential could result in the inhibition of their supporting action on tumor cells and other mesenchymal cells. This could further result in the inhibition of tumor progression and metastasis, reversing chemoradiotherapy sensitivity, and restoring the degree of immune infiltration in tumor areas (Li et al. 2021). Various biomolecules contained in exosomes can mediate the signal transduction of recipient cells after exosome internalization (Kalluri and LeBleu 2020). CDEs are mediators of cell-cell crosstalk between CAFs and intercellular components in the TME. CDE non-coding RNAs are endocytosed by tumor or immune cells, leading to the alteration of downstream signal transduction processes through the regulation of specific gene expression. This ultimately results in phenotypic changes.

Disease-related exosome detection provides valuable bio-signals for early tumor diagnosis, as well as monitoring and assessment of treatment efficacy and prognosis (Liu et al. 2022a, b). However, due to the current technical limitations, the extraction and purification of exosomes as well as the identification of relevant disease signatures is expensive. Moreover, most studies investigating the role of CDE-derived signal molecules in disease diagnosis are still at basic research phases. Relevant clinical studies using these molecules as detection indicators are still nascent. Therefore, the clinical application of liquid biopsy technology based on CDEs remains a challenging diagnostic scheme. The existing gap between basic research evidence and clinical translational applications should be urgently bridged. Furthermore, considering the significant heterogeneity of the signaling markers of CDEs, research regarding tumor diagnosis should investigate a combination of multiple CDE signatures for use in tumor diagnosis. However, scientific research in this area is scarce; thus, large-scale clinical studies involving multiple tumor types are needed to identify and establish the combined diagnostic value of biomarkers that are widely expressed in CDEs of different tumor types (Peng et al. 2021a, b; Liu et al. 2021; Zhu et al. 2020a, b).

The therapeutic applications of exosomes in oncology have also been explored. The exosomes can be divided into two treatment patterns, based on how they affect the TME: first, bioengineered exosomes can be used as carriers of targeted drugs, biological molecules, and immunoregulatory factors to specific tumor areas; second, direct targeting of the synthesis and secretion of cancer-promoting exosomes and the exosome uptake by recipient cells could be optimal strategies for cancer treatment (Bastos et al. 2018; Gilligan and Dwyer 2017). Exosome-based treatment has been applied to different diseases. Some experimental studies have demonstrated potential tumor suppression abilities of these treatments (Kamerkar et al. 2017). However, currently, there is little therapeutic application of CDEs. Numerous studies exploring the mechanisms of reciprocal interactions between CDEs and tumor cells have emphasized their potential therapeutic value. However, these studies were mostly cell- or animal-stage experiments. Hence, it remains unclear whether these biomolecules have a clinically significant therapeutic value. Future studies should explore molecules with therapeutic potential and also utilize existing research data to validate the effects of these molecules. This could provide direct evidence for the development of targeted chemotherapeutic agents and drugs using CDEs.

## Data Availability

Not applicable.

## Abbreviations

CAFs: Cancer-associated fibroblasts
CRC: Colorectal cancer
CDEs: Cancer-associated fibroblast-derived exosomes
EMT: Epithelial–mesenchymal transition
EVs: Extracellular vesicles
HCC: Hepatocellular carcinoma
HNC: Head and neck cancer
ILV: Intraluminal vesicle
MSCs: Mesenchymal stem cells
MVB: Multivesicular bodies
NSCLC: Non-small cell lung cancer
TNBC: Triple-negative breast cancer
PDAC: Pancreatic ductal adenocarcinoma
TME: Tumor microenvironment
α-SMA: Alpha smooth muscle actin
VCAN: Versican
TGF-β: Transforming growth factor-β
FGF: Fibroblast growth factor
PDGF: Platelet-derived growth factor
IL-1: Interleukin-1
IL-6: Interleukin-6
FSP-1: Fibroblast-specific protein-1
SDF-1: Stromal cell derived factor-1
CLCF-1: CAF-derived cardiotrophin-like cytokine factor 1
FAK: Focal adhesion kinase
HIF: Hypoxia-inducible factor
Treg: Regulatory T cell
ICB: Immune checkpoint blockade
FAP: Fibroblast activating protein
NK cells: Natural killer cells
MDSCs: Myeloid-derived suppressor cells
M-MDSCs: Monocytic myeloid-derived suppressor cells
TAMs: Tumor-associated macrophages
VEGF: Vascular endothelial growth factor
ITGB3: Integrin β3
lncRNA: Long non-coding RNA
CSC: Cancer stem cell
THBS2: Thrombospondin 2
NFs: Normal fibroblasts
KRAS: Kirsten rat sarcoma
CTLs: Cytotoxic T lymphocytes.
